# Mitochondrial Oxidative and Nitrosative Stress as a Therapeutic Target in Diseases

**DOI:** 10.3390/antiox10020314

**Published:** 2021-02-20

**Authors:** Montserrat Marí, Anna Colell

**Affiliations:** 1 Department of Cell Death and Proliferation, Institute of Biomedical Research of Barcelona-Spanish National Research Council (IIBB-CSIC), August Pi i Sunyer Biomedical Research Institute (IDIBAPS), 08036 Barcelona, Spain; 2 Department of Cell Death and Proliferation, Institute of Biomedical Research of Barcelona-Spanish National Research Council (IIBB-CSIC), August Pi i Sunyer Biomedical Research Institute (IDIBAPS), Network Center for Biomedical Research in Neurodegenerative Diseases (CIBERNED), 08036 Barcelona, Spain

Mitochondria are fundamental to life. In addition to providing energy, they constitute crucial nodes in multiple signaling pathways that can ultimately decide cell fate. Under physiological conditions, 0.4 to 4% of oxygen consumed by mitochondria is incompletely reduced and leads to the generation of reactive oxygen species (ROS). These chemical moieties, which at low levels are instrumental for several physiological processes and facilitate adaptation to stress via signaling, in excess can be harmful not only to the organelle itself, but also may affect the whole-cell function. Mitochondria have different ways to counter oxidative/nitrosative damage, including a variety of antioxidants, both enzymatic and non-enzymatic mechanisms, of which mitochondrial GSH (mGSH) plays a key role, as described in detail in the review by Marí et al. [1] in this Special Issue. 

Not surprisingly, mitochondrial dysfunction and oxidative/nitrosative stress have been linked to aging and a broad range of human diseases, which has led mitochondria to be the focus of interest of multiple preclinical studies as potential targets for therapeutic intervention. Nonetheless, despite all the efforts made in recent decades, mitochondria-targeted compounds with clinical relevance are missing, indicating that a deeper knowledge of the mechanisms involved is still needed.

The scope of this Special Issue is to give a broad and updated overview of the role of mitochondrial oxidative/nitrosative stress in different pathological conditions. The collection consists of three original research papers and four review articles by renowned experts in the field, providing the reader with specific examples of mitochondrial involvement in metabolic disorders, neurodegenerative diseases, and neuromuscular affections. 

The study by Pittalà et al. [2] exemplifies the involvement of mitochondrial oxidative stress as a significant inducer of selective death of motor neurons in amyotrophic lateral sclerosis (ALS), a neurodegenerative disease that affects nerve cells in the brain and spinal cord, causing loss of muscle control. The study analyses whether oxidative stress in ALS can promote post-translational modifications in VDAC1, the main outer mitochondrial membrane protein known to interact with ALS-related SOD1 mutants. Using the NSC34 cell line expressing human SOD1G93A, they found selective deamidations of asparagine and glutamine of VDAC1, and also over-oxidation of methionine and cysteines in VDAC1 that affect mitochondrial function, and ultimately, cell viability.

Growing evidence also supports the notion that mitochondrial alterations in the brain are key components of the oxidative and nitrosative stress observed in age-dependent neurodegenerative disorders, such as Alzheimer’s disease and its earlier stage, amnestic mild cognitive impairment. These studies are extensively reviewed in the article by Butterfield and Boyd–Kimball [3].

The study by Alegre–Cortés et al. [4] provides deeper insight, analyzing the involvement of the necroptotic machinery in dopaminergic neuron death in Parkinson’s disease (PD), using primary fibroblasts from patients with and without the G2019S leucine-rich repeat kinase 2 (LRRK2) mutation and cells treated with the mitochondrial respiration inhibitor rotenone, a widely used cellular model of PD. Execution of necroptosis, a programmed form of necrosis, has been described to require mitochondrial ROS. Consistent with that, they found that protein executioners of necroptosis are expressed in fibroblasts from PD, but are only activated under rotenone treatment. Rotenone-induced mitochondrial ROS activate necroptosis via RIP1, which is totally abolished by necrostatin-1. Interestingly, in addition to blocking necroptosis, necrostatin-1 treatment disrupts mitophagy, which prevents clearance of rotenone-damaged mitochondria and aggravates rotenone cytotoxicity.

Apart from neurodegeneration, the review article by Cantó–Santos et al. [5] brings a broad vision of the key role of mitochondria in different neuromuscular diseases (NMDs), describing the interrelationship between mitochondrial ROS generation, impaired autophagy, and the increased inflammation observed in many NMDs.

Mitochondrial dysfunction associated with compromised autophagy can also downregulate chondrocyte activity, accelerating the development of osteoarthritis (OA), a degenerative joint disease. The study by Wang et al. [6] has identified Irisin, a cleaved form of fibronectin type III domain-containing 5 (FNDC5), as a compound that may be beneficial for the treatment of OA. They show that intra-articular administration of Irisin reduces inflammation-mediated oxidative stress and extracellular matrix underproduction through engaging mitochondrial biogenesis, mitochondrial dynamics, and the autophagic program.

As stated by Marí et al. [1] the involvement of mitochondrial ROS in the signaling of new prescribed drugs or in other unmet medical needs, such as gender differences or coronavirus disease of 2019 (COVID-19) treatment is still being revealed. Particularly interesting are recent findings showing that mitochondrial function and biogenesis can be regulated by gut microbiota-released metabolites. In this special number, the article by Vezza et al. [7] provides an overview of the existing literature concerning the role of this inter-talk in the onset of obesity and related metabolic disorders such as type 2 diabetes, and discusses the potential mechanisms by which they may become the key to therapeutic strategies to manage these high worldwide prevalence metabolic diseases.

Overall, the articles included in this Special Issue, in addition to presenting the most recent results, illustrate the current challenges and future prospects of research, which may be helpful for the development of future therapies.
